# Imaging-Based Anatomical Characterization of Aortic Aneurysms and Dissections: An Observational Study in a Tertiary Hospital in Bogotá, Colombia

**DOI:** 10.3390/medicina61091558

**Published:** 2025-08-29

**Authors:** Ricardo Miguel Luque Bernal, Angy Carolina Villamil Duarte, Ernesto Fajardo Chavarro, Adriana Urbina, Juan Fernando Cediel Becerra, Sergio Borda, María Paula Cerón Falla, María Andrea Calderón Ardila, Jorge Alberto Carrillo Bayona

**Affiliations:** 1 Anatomy Unit, School of Medicine and Health Sciences, Universidad del Rosario, Bogotá 111221, Colombia; angy.villamil@urosario.edu.co; 2 Department of Vascular Surgery, Hospital Universitario Mayor–Méderi, Bogotá 111411, Colombia; fenarue.fajardo@urosario.edu.co; 3 Grupo INPAC, Facultad de Medicina, Fundación Universitaria Sanitas, Bogotá 110131, Colombia; 4 Applied Biomedical Sciences Research Group (UR BioMed), School of Medicine and Health Sciences, Universidad del Rosario, Bogotá 111221, Colombia; juan.cediel@urosario.edu.co; 5 School of Medicine and Health Sciences, Universidad del Rosario, Bogotá 111221, Colombia; sergio.borda@urosario.edu.co (S.B.); mariap.ceron@urosario.edu.co (M.P.C.F.); jorge.carrillo@urosario.edu.co (J.A.C.B.); 6 Department of Radiology, Hospital Universitario Mayor–Méderi, Bogotá 111411, Colombia; mariaan.calderon@urosario.edu.co

**Keywords:** aortic aneurysm, aortic dissection, Colombia, aorta, risk factors, anatomy

## Abstract

*Background and Objectives:* Aortic aneurysms and dissections are life-threatening vascular disorders with high morbidity and mortality. Enhancing diagnostic and therapeutic strategies requires a precise characterization of their anatomical and clinical features. This study aimed to detail the demographic, clinical, and imaging-based anatomical characteristics of aortic aneurysms and dissections in a cohort of Colombian patients. *Materials and Methods:* We conducted a retrospective, descriptive, observational study at a tertiary hospital in Bogotá, Colombia. Adult patients (≥18 years) with radiologically confirmed aortic aneurysm or dissection on computed tomography angiography (CTA) between 2010 and 2021 were included. Demographic, clinical, and morphological data were extracted. Associations were explored using chi-squared and Mann–Whitney U tests. Multivariate logistic regression was applied to identify independent predictors of in-hospital mortality. *Results:* This study included 539 patients (mean age: 69.3 ± 11.5 years; 53.6% male). Infrarenal abdominal aortic aneurysms were the most frequent (63.7%), with fusiform morphology observed in 92% of cases. Saccular aneurysms were significantly more common in females (*p* = 0.0267) and in non-infrarenal segments (*p* = 0.0162). Among patients with aneurysms, aortic diameter was an independent predictor of mortality (OR = 1.03; 95% CI: 1.01–1.05; *p* < 0.001). No individual variable significantly predicted mortality in dissection cases. *Conclusions:* In this cohort, infrarenal location and fusiform shape predominate. Morphological patterns were associated with sex and anatomical distribution. Although trends were observed, no statistically significant predictors of death were identified in dissection cases. These findings highlight the value of early detection and tailored management and reinforce the importance of generating region-specific data to inform clinical decision making in Latin American settings.

## 1. Introduction

Aortic pathologies, such as aneurysms and dissections, represent a significant clinical and surgical challenge due to their high morbidity and mortality, despite their relatively low prevalence. These conditions require complex diagnostic and surgical resources, as well as prolonged intensive care unit (ICU) stays, resulting in a substantial burden on healthcare systems. Therefore, the early identification of risk factors and timely intervention are critical to reducing complications and improving prognosis [1].

An aortic aneurysm is defined as a dilation exceeding 50% of the normal vessel diameter [2,3]. It has a global prevalence of approximately 4.5 cases per 1000 individuals and ranks as the thirteenth leading cause of death in Western countries [4,5]. Thoracic aortic aneurysms, in particular, have an incidence ranging from 5 to 10 cases per 100,000 people, with rupture-associated prehospital mortality rates of 22%, rising to 34% within the first 30 days [6,7]. Morphologically, aneurysms are classified based on their location within the aorta (ascending, arch, descending thoracic, suprarenal abdominal, and infrarenal) and by shape: fusiform when the vessel is uniformly dilated, and saccular when a focal dilation is observed adjacent to a segment of normal-diameter aorta [8,9].

Aortic dissection, on the other hand, is characterized by a pathological separation of the tunica intima and media, usually resulting from medial necrosis [10]. Its incidence is estimated at 2 to 3 cases per 100,000 individuals, with a mortality rate of up to 50% within the first 48 h. Traditionally, dissections are classified using the Stanford (types A and B) or DeBakey (types I, II, and III) systems. More recently, the TEM classification system has been proposed, incorporating not only anatomical type and location but also the entry point of the dissection and the presence of malperfusion, enabling a more nuanced assessment of prognosis and treatment strategies [11,12,13,14].

While aortic aneurysms and dissections are well documented in international literature, there remains a substantial gap in the clinical and morphological characterization of these entities in Latin American populations, particularly in Colombia. Most available data are derived from populations with distinct demographic, clinical, and genetic characteristics, limiting their applicability to the Colombian context. Although isolated studies in Medellín and Bogotá have reported on the prevalence of abdominal aortic aneurysms [1,13], systematic analyses of shape, location, and clinical correlates of aortic aneurysms and dissections in Colombian patients are lacking.

In this context, the objective of this study is to provide a clinical and morphological characterization of aortic aneurysms and dissections in a cohort of patients treated at a high-complexity hospital in Bogotá, Colombia, over a 12-year period.

## 2. Materials and Methods

A retrospective, observational, and descriptive study was conducted at Hospital Universitario Mayor–Méderi, Bogotá, Colombia. All Colombian patients aged ≥18 years who underwent contrast-enhanced computed tomography angiography (CTA) between January 2010 and December 2021 were considered for inclusion.

**Figure 1:**
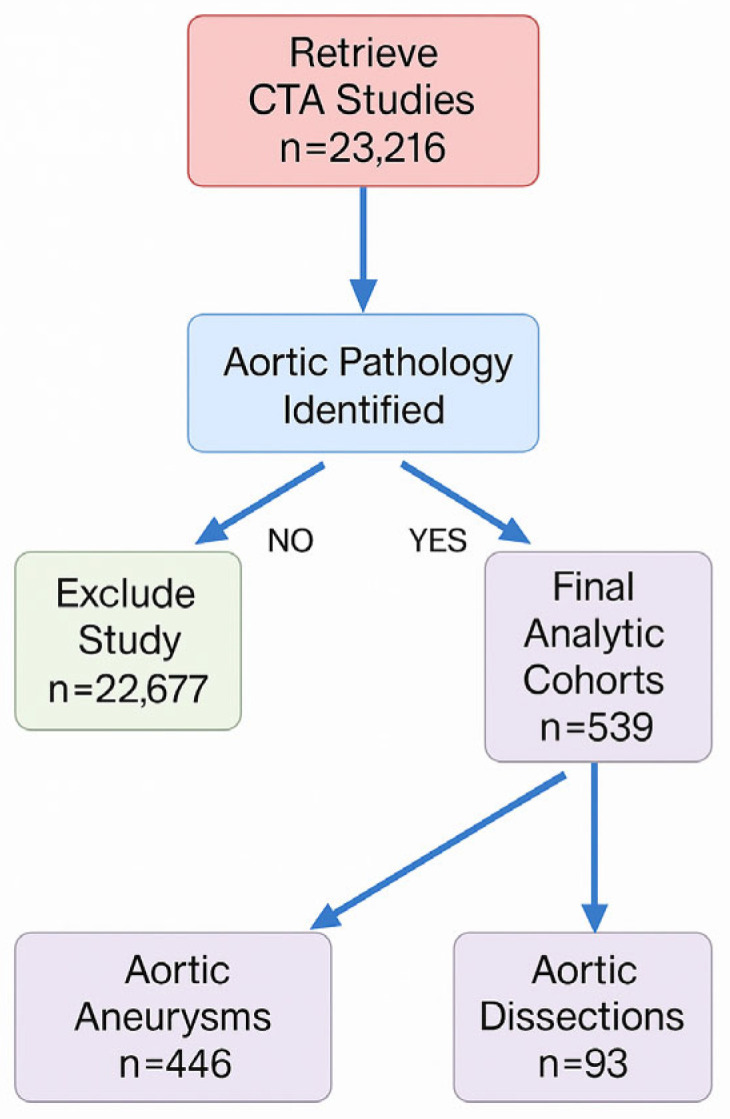
Study flow diagram. All contrast-enhanced CTA examinations performed in adults between January 2010 and December 2021 (n = 23,216) were screened manually. After excluding 22,677 scans with no aortic disease, 539 studies with aortic pathology remained: 446 aneurysms (abdominal or thoracic) and 93 dissections.

A total of 23,216 radiology reports were manually reviewed by the research team. Only reports issued by board-certified radiologists that included a confirmed diagnosis of aortic aneurysm or dissection were included. Reports lacking explicit confirmation were excluded. Morphological and clinical data were extracted directly from the radiology reports and the electronic medical records; no independent review of the CTA images was performed. While this approach reflects real-world clinical workflows and ensures expert interpretation, it precludes formal inter-observer agreement analysis and may introduce classification bias at the report level. The screening process is illustrated in Figure 1.

The final sample was a convenience cohort drawn from institutional imaging archives. For each confirmed case, demographic, clinical, and morphological variables were collected, including age, sex, anatomical location, aneurysm morphology (fusiform, saccular, or mixed), maximum diameter, and relevant comorbidities (hypertension, diabetes mellitus, smoking history, prior thoracic surgery, vasculitis, congenital heart disease, connective tissue disorders, and congenital malformations).

Aneurysms were identified when segment-specific size thresholds were met on CTA: ≥3.0 cm for the abdominal aorta, ≥5.0 cm for the ascending thoracic aorta, and ≥4.0 cm for the descending thoracic aorta. Aortic dissections were diagnosed based on the presence of a double-lumen appearance with an intimal flap or penetrating aortic ulcer consistent with dissection.

The aorta was segmented into five anatomical regions for morphological analysis: (i) ascending aorta, (ii) aortic arch, (iii) descending thoracic aorta, (iv) suprarenal abdominal aorta, and (v) infrarenal abdominal aorta. Aneurysms were assigned to a region based on radiology report descriptions. Dissections were classified according to the DeBakey system, which distinguishes between type I (ascending with extension), type II (ascending only), and type III (distal to left subclavian, subclassified as IIIa or IIIb depending on abdominal extension). This dual classification system allowed for detailed comparisons across aortic segments.

Cases with missing or indeterminate morphological data were excluded. Aneurysms were also categorized by timing of diagnosis: newly diagnosed cases were identified during the index imaging (whether symptomatic or incidental), while previously known cases had a documented history prior to the current episode. Presence of rupture was recorded as a separate variable. Vital status at the end of the follow-up period was classified into three mutually exclusive categories: (1) alive, (2) deceased due to aneurysm/dissection-related causes, and (3) deceased from unrelated or unknown causes. In addition to hospital records, public death registries were reviewed to identify and classify out-of-hospital deaths, ensuring a more comprehensive ascertainment of mortality outcomes.

For every patient with aortic dissection, involvement of the main visceral arteries—the coeliac trunk, superior mesenteric artery (SMA), inferior mesenteric artery (IMA), and both renal arteries—was abstracted directly from the CTA report. A branch was considered involved when the radiologist described (i) origin from the false lumen, (ii) intraluminal thrombosis, or (iii) radiological signs of malperfusion or ischaemia. If the report stated that the artery arose from the true lumen and no perfusion abnormality was mentioned, the vessel was coded as not involved. When involvement was documented, the specific branches affected were recorded (coeliac, SMA, IMA, right renal, left renal, or bilateral), enabling subgroup analysis of visceral malperfusion patterns.

Descriptive statistics included means, standard deviations, medians, and interquartile ranges for continuous variables, and frequencies and percentages for categorical variables. Associations between categorical variables were assessed using chi-squared or Fisher’s exact tests. Continuous variables were compared using the Mann–Whitney U test after evaluating distribution with the Shapiro–Wilk test.

A multivariate binary logistic regression was performed to identify independent predictors of mortality (non-survival). Variables with *p* < 0.25 in univariate analyses were included in the initial model; stepwise backward elimination was applied. Multicollinearity was assessed using the variance inflation factor (VIF). Model calibration and discrimination were evaluated using the Hosmer–Lemeshow test and area under the ROC curve, respectively. Results were reported as crude and adjusted odds ratios (ORs) with 95% confidence intervals (CIs).

Additionally, a multinomial logistic regression model was constructed to assess predictors of mortality across the three defined outcomes (alive, death due to aneurysm/dissection, and death from unrelated causes), allowing for a more granular understanding of risk factors.

All statistical analyses were conducted using R software (version 2023.12.1+402). Figures were created in Microsoft PowerPoint and refined with the assistance of the ChatGPT AI tool (OpenAI, GPT-4o, 2024 version).

The study protocol was approved by the Research Ethics Committee of Universidad del Rosario. As it was based exclusively on secondary, anonymized data, it was classified as minimal risk under Resolution 8430 of 1993 by the Colombian Ministry of Health and conducted in accordance with the Declaration of Helsinki.

## 3. Results

A total of 23,216 medical records of patients who underwent CT angiography (AngioCT) between 2010 and 2021 at Hospital Universitario Mayor–Méderi were reviewed. As a result, 446 patients were identified with a diagnosis of aortic aneurysm and 93 with aortic dissection.

### 3.1. Aortic Aneurysm

Among patients with aneurysms, 238 (53.4%) were male and 208 (46.6%) female. The average age was 73.8 years for men and 75.1 years for women. Hypertension was the most frequent comorbidity (74.5%), followed by diabetes mellitus (11.9%) and history of thoracic surgery (0.7%) (Table 1). Of the 443 patients with aortic aneurysms, 89 cases (20.1%) were classified as recently diagnosed, identified during a clinical encounter either due to symptoms or as incidental findings. The remaining 354 cases (79.9%) corresponded to previously known aneurysms that had been documented in the medical record prior to the index event.

**Figure 2:**
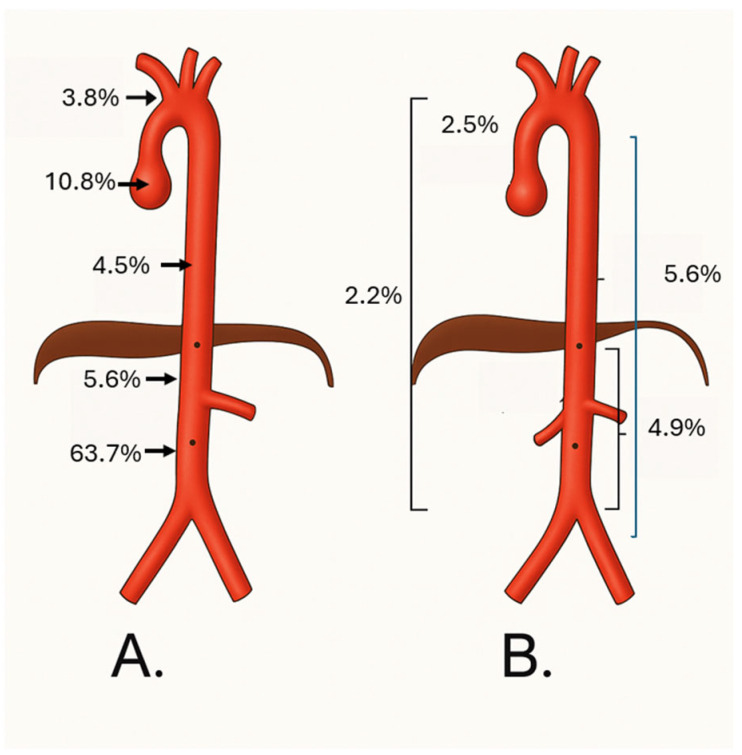
Segmental Distribution of Aortic Aneurysms in Patients Treated at a High-Complexity University Hospital. (**A**) Schematic representation of the relative frequency of aneurysms by anatomical aortic segment. (**B**) Comparative distribution in an independent cohort. Percentages indicate the proportion of aneurysms located in each region, highlighting the predominance of infrarenal aneurysms in panel (**A**).

**Figure 3:**
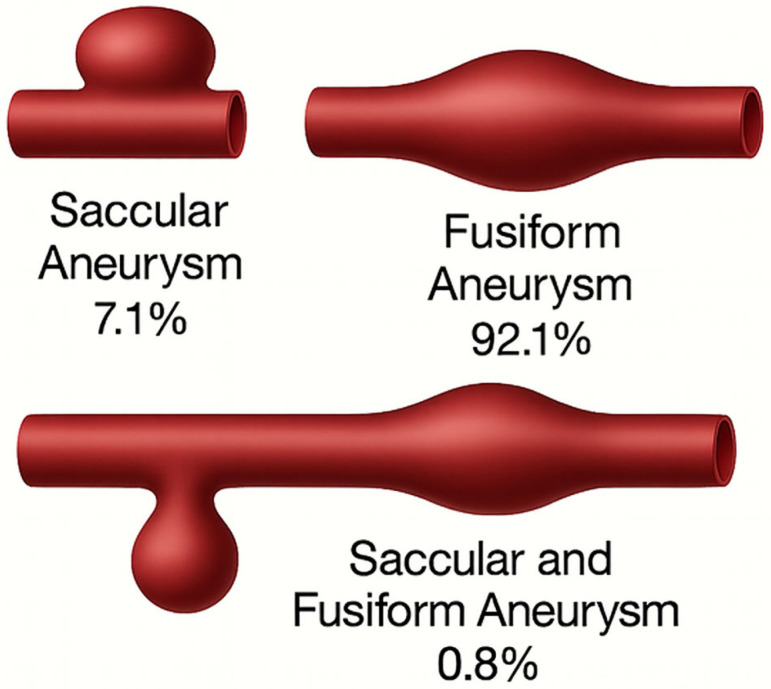
Schematic Representation of Morphological Types of Aortic Aneurysms. The three morphological types identified are illustrated: saccular (7.1%), fusiform (92.1%), and mixed saccular-fusiform (0.8%). The image depicts the typical anatomical configuration of each type, based on morphological findings from the evaluated cohort.

Aneurysms were predominantly located in the infrarenal abdominal aorta (63.6%), followed by the ascending aorta (10.8%) and descending thoracic aorta (4.7%) (Figure 2). Among the 280 patients with recorded aneurysm morphology, 91.8% had fusiform aneurysms, 7.1% saccular, and 1.1% mixed morphology (Figure 3).

Bivariate analysis showed no significant difference between males and females in the overall presence of aneurysms (*p* = 0.2735). No significant relationship was identified between aneurysm morphology and diabetes mellitus (*p* = 0.417). However, saccular aneurysms were significantly more frequent in females (*p* = 0.0267). There was also a significant association between aneurysm morphology and anatomical location: saccular aneurysms were more common outside the infrarenal region (*p* = 0.0162). This difference was particularly marked in the aortic arch, where 72% of aneurysms were saccular, compared to the predominance of fusiform aneurysms in other regions of the aorta, a highly significant difference (*p* = 1.57 × 10^−15^). Furthermore, a significant association was found between the presence of a bicuspid aortic valve and aneurysms in the ascending aorta, as all patients with a bicuspid valve presented aneurysms in this region (*p* = 0.0000051) (Table 2).

Multivariate logistic regression analysis identified key predictors of mortality in patients with aortic aneurysms. Age, analyzed in 5-year increments, was significantly associated with increased risk of death (adjusted OR = 1.23; 95% CI: 1.01–1.48; *p* = 0.036). Similarly, the maximum aortic diameter was an independent predictor of mortality across two models. In the global mortality model, each additional millimeter in aortic diameter increased the odds of death by 2% (adjusted OR = 1.02; 95% CI: 1.01–1.03; *p* < 0.001), while in the aneurysm-related death subgroup, the risk rose by 3% per millimeter (adjusted OR = 1.03; 95% CI: 1.01–1.05; *p* < 0.001). Prior thoracic surgery showed a trend toward increased mortality, although it did not reach conventional statistical significance (adjusted OR = 2.78; 95% CI: 0.99–7.83; *p* = 0.053) (Table 3).

### 3.2. Aortic Dissection

Among the 93 patients with aortic dissection, 51 (54.8%) were male and 42 (45.2%) female, with a mean age of 67.1 and 66.5 years, respectively. Hypertension was present in 79.6% of cases, diabetes mellitus in 12.9%, prior thoracic surgery in 8%, and 1.1% were diagnosed with Marfan syndrome (Table 1).

Dissections were anatomically classified according to the DeBakey classification, with type III being the most frequent (53.8%), followed by type I (33%) and type II (12.9%) (Table 4).

Among the 93 patients with aortic dissection, 19 cases (20%) showed visceral-branch involvement on the index CTA. The renal arteries were the most frequently affected (11%), followed by the superior mesenteric artery (7%) and the coeliac trunk (4%); inferior mesenteric artery compromise was not observed. In six patients (6%), more than one visceral branch was involved, typically a combination of renal and mesenteric vessels. Visceral malperfusion was significantly more common in acute dissections than in chronic cases (25% vs. 5%; *p* = 0.018), but it was not associated with DeBakey type (*p* = 0.41). Mortality was higher in patients with visceral involvement (37% vs. 17%; adjusted OR = 2.34, 95% CI 1.01–5.45, *p* = 0.047), independent of age and maximum aortic diameter.

Bivariate analysis did not reveal a significant association between the DeBakey classification and diabetes mellitus (*p* = 0.113). In contrast, a significant relationship was found between history of thoracic surgery and dissection type, with a higher proportion of previous interventions in patients with type II dissection (*p* = 0.004). No significant associations were observed between hypertension and dissection location (*p* = 0.913) or between age (<60 vs. ≥60 years) and dissection type (*p* = 0.204), although there was a trend for older patients to present more frequently with type III dissection.

Finally, factors associated with survival in patients with aortic dissection were explored using a logistic regression model including age, sex, DeBakey classification, diabetes mellitus, and hypertension. None of these variables reached statistical significance (*p* > 0.05). However, type III dissection showed a trend toward a higher probability of survival (OR ≈ 2.95; *p* = 0.157). Conversely, both diabetes and hypertension were associated with lower survival probability (*p* = 0.134 and *p* = 0.212, respectively), though not statistically significant. Age (*p* = 0.275) and sex (*p* = 0.286) also showed.

Of the 12 patients classified as DeBakey II, 8 cases (67%) were de novo, with no record of previous thoracic-aortic surgery, whereas 4 cases (33%) occurred as late sequelae of repaired DeBakey I (Stanford A) dissections, all of which had undergone prior ascending-aorta grafting procedures (e.g., Bentall or tube graft replacement). Consistent with this finding, prior thoracic surgery remained significantly over-represented in the type II group compared with other dissection types (*p* = 0.004).

## 4. Discussion

Aortic pathologies associated with acute aortic syndrome have been extensively described in the international literature; however, there is limited evidence regarding their characteristics in Latin American populations, particularly in Colombia. This study helps bridge that gap by identifying clinical and morphological patterns unique to a hospital-based cohort in a high-complexity context.

### 4.1. General Clinical Aspects

This study, one of the most comprehensive conducted in Colombia on aortic pathologies, provides relevant data on the clinical and morphological characterization of aortic aneurysms and dissections. From over 23,216 imaging studies reviewed, 446 cases of aneurysm and 93 of dissection were identified. The mean age in both groups exceeded 65 years, consistent with international literature, and reinforces the close association between vascular aging and aortic disease [15].

### 4.2. Aortic Aneurysm: Clinical and Morphological Characteristics

Aneurysms were slightly more common in men (53.4%), although the difference was smaller than reported in prior studies, where male predominance can reach up to 4:1 [5]. This could reflect a selection bias in the hospital-based sample, potentially influenced by higher rates of medical follow-up and lower mortality among women. Arterial hypertension was the most frequent comorbidity (74.5%), followed by diabetes mellitus (11.9%), similar to national prevalence data in older adults [16]. Interestingly, literature has suggested that diabetes might act as a protective factor against aneurysm development, though no significant association was observed in this study [17].

Regarding morphology, 91.8% of aneurysms were fusiform, 7.1% saccular, and 1.1% mixed, consistent with previous reports. A strong association was found between saccular morphology and non-infrarenal locations (*p* = 0.0162), particularly in the aortic arch, where 72% of aneurysms were saccular (*p* < 1.57 × 10^−15^), suggesting a possible underrecognized anatomical-clinical pattern. A significant association was also observed between bicuspid aortic valve and ascending aorta aneurysms (*p* = 0.0000051), supporting its consideration as a risk marker for this location [18,19].

Aneurysm diameter was closely related to mortality, acting as a prognostic factor. Larger aneurysms were associated with poorer survival (*p* < 0.001), especially those exceeding 66 mm in diameter, consistent with existing literature. Age, in contrast, was not significant in the multivariate model, underscoring the importance of aneurysm size over chronological age in determining intervention [20].

### 4.3. Aortic Dissection: Profile and Patterns

Dissections were more frequent in men (54.8%) and occurred at younger ages compared to aneurysm cases, with 11.8% diagnosed before age 50. This aligns with studies suggesting an earlier onset, especially among patients with genetic predisposition or Marfan syndrome. Hypertension was prevalent (79.6%), and prior thoracic surgery was significantly more common in patients with type II dissection (*p* = 0.004), indicating a possible relationship between previous surgical interventions and structural changes in the proximal aorta [21,22].

The DeBakey classification revealed a predominance of type III dissections (53.8%), differing from literature expectations where types I and II represent about two-thirds of cases. This may reflect selection bias, as only patients with imaging-confirmed diagnoses were included, potentially excluding those with fulminant proximal dissections who died before hospital arrival.

Although no variables reached statistical significance in the multivariate survival model for dissection, type III dissections showed a trend toward better prognosis (OR ≈ 2.95). In contrast, both diabetes and hypertension were associated with lower survival probabilities, though without statistical significance. These trends suggest that larger sample sizes may confirm these associations [22].

This study has several limitations. First, its retrospective design inherently limits the control over data quality and completeness. Second, as it was conducted in a single tertiary hospital, the findings may not be fully generalizable to other populations or healthcare settings. Additionally, due to the nature of the available records, it was not possible to determine specific causes of death in all cases, particularly when deaths were classified as unrelated or of unknown origin. Finally, although this study spanned over 11 years, the low prevalence of aortic aneurysms and dissections restricted the statistical power for certain subgroup analyses and prevented more robust conclusions in some specific categories.

## 5. Conclusions

This study highlights the relevance of morphological analysis in diagnosing and prognosticating aortic pathologies. In aneurysms, both diameter and anatomical location were associated with clinical outcomes, while anatomical classification remained key in dissections. The observed differences compared to international data underscore the need for region-specific evidence in Latin American populations, enabling more contextualized decision making. Incorporating these findings into clinical protocols may help optimize management and reduce associated mortality.

## Figures and Tables

**Table 1:** Demographic and Clinical Characteristics of Patients with Aortic Aneurysm and Dissection.

		Aneurysm		Dissection	
		No.	%	No.	%
	Total patients	446	100	93	100
Age	<50 years	5	1.1	11	11.8
	50–59	24	5.4	16	17.2
	60–69	103	23.1	22	23.7
	70–79	167	37.4	27	29.0
	≥80	143	32.1	17	18.3
	Unspecified	4	0.9	0	0.0
Sex	Male	238	53.4	51	54.8
	Female	208	46.6	42	45.2
Comorbidities	Hypertension	332	74.4	74	79.6
	Diabetes mellitus	53	11.9	12	12.9
	Prior thoracic surgery	3	0.7	8	8.6
	Marfan syndrome	0	0.0	1	1.1
	Vasculitis	2	0.4	0	0.0
	Thoracic trauma	0	0.0	1	1.1
	Congenital heart disease	3	0.7	1	1.1
	No comorbidities	87	19.5	21	22.6

Absolute and relative frequencies of patients diagnosed with aneurysm (n = 446) or aortic dissection (n = 93), stratified by age, sex, and comorbidities. Hypertension was the most frequent comorbidity in both groups, while prior thoracic surgery, genetic syndromes, and traumatic history were more prevalent in dissection cases.

**Table 2:** Bivariate Analysis of Clinical, Morphological, and Demographic Variables in Aneurysm Patients.

	Nombre	Characteristic		*p*-Value
Diabetes and aneurysm location	Characteristic	Infrarenal aneurysm Yes	Infrarenal aneurysm No	0.0153
	Diabetes: Yes	37 (13.1%)	9 (3.2%)	
	Diabetes: No	246 (87.2%)	153 (96.8%)	
Thoracic surgery and location	Characteristic	Ascending aorta	Non-ascending aorta	0.0660
	Thoracic Surgery: Yes	7 (12.5%)	23 (87.5%)	
	Thoracic Surgery: No	49 (11.8%)	366 (88.2%)	
Sex and thoracic surgery	Characteristic	Surgery	No surgery	1.06 × 10^−6^
	Male	25 (10.5%)	213 (89.5%)	
	Female	5 (2.4%)	202 (97.6%)	
Sex and survival	Characteristic	Yes	No	0.0956
	Male	211 (88.7%)	27 (11.3%)	
	Female	193 (93.2%)	14 (6.8%)	
Hypertension and location	Characteristic	Infrarenal	Other locations	0.0905
	Yes	203 (61.3%)	128 (38.7%)	
	No	80 (70.2%)	34 (29.8%)	
Sex and aneurysm morphology	Characteristic	Saccular	Fusiform	0.0267
	Male	6 (4.0%)	143 (96.0%)	
	Female	14 (10.9%)	114 (89.1%)	
Location (infrarenal vs. other) and morphology	Characteristic	Saccular	Fusiform	0.0162
	Infrarenal aorta	9 (4.7%)	182 (95.3%)	
	Other aortic segments	11 (12.8%)	75 (87.2%)	
Location (arch vs. other) and morphology	Characteristic	Saccular	Fusiform	1.57 × 10^−15^
	Aortic arch	8 (72.7%)	3 (27.3%)	
	Other aortic segments	12 (4.5%)	254 (95.5%)	
Bicuspid aortic valve and location	Characteristic	Ascending aorta	Other aortic segments	5.1 × 10^−6^
	Yes	4 (100%)	0 (0%)	
	No	43 (11.6%)	329 (88.4%)	

*p*-values from chi-squared tests for categorical variables. Statistically significant associations (*p* < 0.05) are highlighted with directional trends.

**Table 3:** Multivariate logistic regression analysis of predictors of mortality in patients with aortic aneurysm. Age was analyzed in 5-year increments. Maximum aortic diameter was assessed both for overall mortality and for deaths attributable specifically to aneurysm-related causes.

Variable	Adjusted OR	95% CI	*p*-Value
Age (per 5-year increment)	1.23	1.01–1.48	0.036
Maximum aortic diameter (per mm)–overall mortality	1.02	1.01–1.03	<0.001
Maximum aortic diameter (per mm)–aneurysm-related death	1.03	1.01–1.05	<0.001
Prior thoracic surgery	**2.78**	**0.99–7.83**	**0.053**

Model diagnostics (global mortality model): N = 446; pseudo-R^2^ = 0.102; likelihood ratio χ^2^ = 23.5 (*p* = 3.99 × 10^−5^); Hosmer–Lemeshow *p* > 0.05; all variance inflation factors (VIFs) < 2; AUC = 0.75.

**Table 4:** Distribution of Aortic Dissection Patients by Age Group and DeBakey Classification.

Age	Type I	Type II	Type III	Total
<55 years	8 (8.6%)	3 (3.2%)	5 (5.4%)	16 (17.2%)
55–59	1 (1.1%)	3 (3.2%)	7 (7.5%)	11 (11.8%)
60–64	2 (2.2%)	1 (1.1%)	4 (4.3%)	7 (7.5%)
65–69	6 (6.5%)	1 (1.1%)	8 (8.6%)	15 (16.1%)
70–74	7 (7.5%)	0 (0.0%)	9 (9.7%)	16 (17.2%)
75–79	2 (2.2%)	1 (1.1%)	8 (8.6%)	11 (11.8%)
≥80	5 (5.4%)	3 (3.2%)	9 (9.7%)	17 (18.3%)
Total	31 (33.3%)	12 (12.9%)	50 (53.8%)	93 (100%)

Type III dissection was most frequent (53.8%), followed by type I (33.3%) and type II (12.9%). Most type III cases occurred in patients aged ≥65 years.

## Data Availability

The data presented in this study are not publicly available due to ethical restrictions. Access to the datasets is limited in order to protect patient confidentiality, as per the approval granted by the Institutional Ethics Committee of Universidad del Rosario and the Hospital Universitario Mayor–Méderi. Further inquiries can be directed to the corresponding author.

## Abbreviations

The following abbreviations are used in this manuscript:

AA	Aortic Aneurysm
AD	Aortic Dissection
CTA	Computed Tomography Angiography
ICU	Intensive Care Unit
OR	Odds Ratio
CI	Confidence Interval
ROC	Receiver Operating Characteristic
VIF	Variance Inflation Factor
